# Plasma Proteomic Profiling Identifies Biomarkers and Therapeutic Targets for Predicting Osteoporosis Risk in Patients with Inflammatory Bowel Disease

**DOI:** 10.5152/tjg.2026.26320

**Published:** 2026-08-04

**Authors:** Min Yue, Xiaohua Ye, Zhenhe Jin, Kexin Ye, Chengwei Xu, Tianyu Zhou, Zhe Shen

**Affiliations:** 1 Department of Gastroenterology, First Affiliated Hospital, Zhejiang University School of Medicine, Hangzhou, China; 2 Department of Gastroenterology, Affiliated Jinhua Hospital, Zhejiang University School of Medicine, Jinhua, China

**Keywords:** IBD–osteoporosis multimorbidity, machine learning, multistate modeling, plasma proteomic, UK Biobank

## Abstract

**Background/Aims:**

Inflammatory bowel disease (IBD) is a systemic disorder that affects not only the gastrointestinal tract but also extraintestinal organs and is associated with a significantly increased risk of osteoporosis. This study aims to investigate the association between plasma proteomic profiles and the subsequent risk of osteoporosis in patients with IBD.

**Materials and Methods:**

Using data from 52 953 participants in the UK Biobank Pharma Proteomics Project, overlapping plasma proteins associated with both IBD and osteoporosis using Cox regression analysis were identified. Among these proteins, those with high predictive importance for IBD–osteoporosis multimorbidity were selected using machine learning and multistate modeling. Single-cell–type expression analysis was conducted to evaluate the cell type–specific enrichment of the candidate predictive protein-coding genes in the intestine.

**Results:**

Over a median follow-up of 13.6 years, 339 proteins were significantly associated with IBD and 183 with osteoporosis, with 104 proteins overlapping between the 2 conditions. Machine learning and multistate modeling narrowed these down to 8 candidate predictive proteins (PLAUR, TNFRSF10A, SPINK1, HLA_E, CHGA, SPP1, TNFRSF10B, and IGFBP2) for IBD–osteoporosis multimorbidity. Exploratory single-cell expression analysis of 5 available candidate genes characterized their intestinal expression patterns, providing supportive biological insights.

**Conclusion:**

The study delineates the proteomic signatures associated with IBD–osteoporosis multimorbidity, identifies specific proteins associated with both conditions, highlights key molecular players linking the diseases, and identifies both established and potentially novel candidate biomarkers.

**Main Points**

- Inflammatory bowel disease (IBD) is associated with a significantly increased risk of osteoporosis.
- This study delineates the proteomic signatures associated with IBD–osteoporosis multimorbidity.
- This study identifies key molecular players linking IBD and osteoporosis and highlights both established and potentially novel candidate biomarkers.

## Introduction

Inflammatory bowel disease (IBD), encompassing Crohn’s disease (CD) and ulcerative colitis (UC), is a chronic and progressive disorder that has become a major global health concern. Population aging in Western countries is contributing to the increasing prevalence of IBD, whereas the ongoing epidemiological transition in Asia and Latin America is expected to drive a substantial rise in incidence, contributing to an estimated 7 million affected individuals worldwide.^1^^-^^3^ This trend places a substantial burden on healthcare systems worldwide through both direct medical costs and indirect costs, including reduced productivity, increased disability, and impaired quality of life.^4^^,^^5^

IBD is widely recognized as a systemic condition that affects not only the gastrointestinal tract but also extraintestinal organs, including bone, joints, skin, and eyes.^6^ Numerous studies have reduced bone mineral density (BMD) in patients with IBD, with prevalence estimates ranging from 12% to 70%.^7^^-^^9^ A meta-analysis reported a relative risk of fracture of 1.41 (95% CI: 1.26-1.56) in patients with IBD, corresponding to a 40% higher risk than that in the general population.^10^ Although established risk factors such as malnutrition, malabsorption, calcium/vitamin D deficiency, and corticosteroid treatment contribute to osteoporosis in patients with IBD, the inflammatory process itself is increasingly recognized as a key mechanism underlying disease-related bone loss.^11^^-^^13^ Indeed, the pathogenesis of low BMD in patients with IBD is complex, multifactorial, and remains incompletely understood.

This study aims to investigate the association between plasma proteomic profiles and the subsequent risk of osteoporosis in patients with IBD. Using data from the UK Biobank, baseline calcaneal BMD and T scores were compared between individuals with different IBD subtypes and healthy controls. Subsequently, in a prospective longitudinal subcohort of 52 953 participants with available Olink plasma proteomic profiles, protein-level changes preceding the diagnosis of IBD-related osteoporosis were analyzed and the multistate transition to IBD–osteoporosis multimorbidity was modeled. Based on these findings, a machine learning model and a multistate model were developed to facilitate early identification and prognostic stratification. This research not only advances the understanding of IBD-related osteoporosis but also helps clarify the potential role of plasma proteomics in its pathogenesis.

## Materials and Methods

### Study Design and Data Source

This investigation leveraged the UK Biobank, a large-scale prospective cohort comprising more than 500 000 individuals aged 37 to 73 years at enrollment. Between 2006 and 2010, participants were recruited from 22 assessment centers across England, Scotland, and Wales. Baseline BMD was compared between participants with different IBD subtypes and healthy controls. This analysis included 445 064 participants with complete demographic and BMD data. Subsequently, a prospective longitudinal study was conducted to evaluate incident IBD, incident osteoporosis, and multistate transitions to IBD–osteoporosis multimorbidity. Follow-up continued until October 31, 2022, for participants in England; May 31, 2022, for those in Wales; and August 31, 2022, for those in Scotland. This longitudinal analysis was restricted to a subcohort of 52 953 participants with available Olink plasma proteomic profiles at enrollment. Eligible participants were required to maintain consent throughout follow-up, have no history of IBD or osteoporosis at baseline, and have complete covariate and proteomic data. The UK Biobank study was approved by the Ethics Committee of North West Multi-centre Research (approval number: 299116, date: June 29, 2021). Because this study was a secondary analysis of deidentified data conducted under an approved application, additional ethical approval and informed consent were not required.

### Disease Ascertainment

Incident cases were identified using the International Classification of Diseases-10 (ICD-10) diagnostic codes. Diagnostic information was obtained from primary care and hospital inpatient records. IBD was defined by ICD-10 codes K50 (CD), K51 (UC), and K52 (undetermined colitis). Osteoporosis was defined by ICD-10 codes M80 (osteoporosis with pathological fracture) and M81 (osteoporosis without current pathological fracture) (Supplementary Table 1).

### Baseline Characteristics

Demographic and clinical variables collected at enrollment included age, sex, ethnic status, body mass index (BMI), Townsend deprivation index (TDI), education level, smoking status, alcohol consumption, physical activity, hypertension, lipidemias, and diabetes. In addition, bone density measurements and T scores were obtained for all participants.

### Proteomic Profiling

Venous blood samples were collected in EDTA tubes at baseline. Plasma was isolated by centrifugation (2500 *g* for 10 minutes at 4°C), aliquoted, and stored at −80°C until analysis. Detailed sample collection and processing procedures are described in the UK Biobank protocol.^14^

Between April 2021 and February 2022, plasma samples from 54 306 participants were transported on dry ice to Olink Analysis Service (Sweden) for high-throughput proteomic analysis. The Olink Explore™ Proximity Extension Assay platform quantified 2920 proteins using 4 384-well panels (cardiometabolic, inflammation, neurology, and oncology), with each panel comprising 4 abundance blocks for every 96 samples.^15^ Stringent quality control procedures were implemented according to published guidelines (biobank. ndph. ox. ac. uk/ukb/ukb/docs/ PPP_Phase_1_QC_dataset_companion_doc.pdf), confirming the absence of systematic batch effects.

### Baseline Bone Mineral Density Assessment

Baseline BMD and T scores were estimated by quantitative ultrasound measurements of the heel bone using the Sahara Clinical Bone Sonometer. The device estimates BMD from the speed of sound and broadband ultrasound attenuation (BUA), which are combined to generate the Quantitative Ultrasound Index (QUI). BMD and T scores are then derived from the QUI relative to a sex-matched healthy young adult reference population.

### Statistical Methodology

#### Protein–Disease Association Analysis:

Cox proportional hazards regression was used to examine the associations between individual proteins and disease incidence, with adjustment for age, sex, ethnicity, BMI, TDI, education level, smoking status, alcohol consumption, physical activity, hypertension, lipidemias, and diabetes. Hazard ratios were estimated to quantify these associations. To account for multiple testing, statistical significance was defined using a Bonferroni-adjusted threshold (*P* < .05/2920). Significant proteins were categorized as disease specific or shared between the 2 conditions. Analyses were performed using the “survival” package (v3.8.3) in R.

#### Pathway Enrichment Analysis:

Functional enrichment analysis was performed using the clusterProfiler package (v4.14.3) to identify overrepresented biological pathways among comorbidity-associated proteins. Gene Ontology (GO) enrichment analysis examined 3 ontological categories: biological process (BP), cellular component (CC), and molecular function (MF). Kyoto Encyclopedia of Genes and Genomes (KEGG) pathway analysis mapped proteins to established signaling pathways and metabolic networks. Statistical significance was determined using the hypergeometric test with Benjamini–Hochberg correction (adjusted *P* < .05). Transcription factor enrichment analysis was performed using the enrichR package (v3.4), screening curated databases (ENCODE, ChEA, and TRANSFAC) to identify candidate upstream regulators whose target genes significantly overlapped with the protein list.

Protein–protein interaction networks were constructed using the STRING database (version 12.0) with the default confidence parameters (minimum interaction score, 0.400), incorporating experimentally validated interactions and functional associations from multiple sources.^16^ Networks were visualized using Cytoscape (version 3.9.1), and topological parameters including node degree and betweenness centrality were computed to characterize network architecture.^17^ Hub proteins were identified using the cytoHubba plugin and its Maximal Clique Centrality algorithm, which ranks nodes according to their participation in maximal cliques and has been shown to perform well in identifying biologically relevant hub proteins.^18^

#### Predictive Modeling:

Machine learning using the Light Gradient Boosting Machine algorithm was performed to evaluate proteins jointly associated with both conditions. The dataset was partitioned into training (80%) and testing (20%) sets. To address class imbalance, the Synthetic Minority Over-sampling Technique was applied during model training. Hyperparameter tuning was performed using Bayesian optimization to maximize predictive performance.^19^ Model generalizability and stability were evaluated using 5-fold cross-validation, with the mean area under the receiver operating characteristic curve reported across all folds. To interpret the contribution of individual proteins to model predictions, SHAP values were calculated to quantify the impact of each feature on prediction outcomes. All computational analyses were performed in R version 4.4.2 (R Foundation for Statistical Computing; Vienna, Austria)using the lightgbm (v4.6.0), xgboost (v1.7.10.1), glmnet (v4.1-8), and randomForest (v4.7-1.2) packages. 

#### Multistate Transition Analysis:

A multistate framework was used to model disease trajectories across 3 states: (1) disease free, (2) a single disease (IBD or osteoporosis), and (3) comorbidity. The framework incorporated 5 transition pathways: healthy-to-single disease (transitions 1-2) and single-to-dual disease (transitions 3-4). Concurrent diagnosis was defined as the onset of both diseases within 0.5 years.

Transition-specific Cox models were fitted using the mstate package (v0.3.3), with adjustment for demographic and anthropometric factors. The proportional hazards assumptions was assessed using Schoenfeld residuals. Stratified Cox models [“strata(trans)”] were used to estimate protein effects across transitions while allowing transition-specific baseline hazards.^20^

Kaplan–Meier analysis was used to generate survival curves, and groups were compared using the log-rank tests. The Aalen–Johansen estimator was used to generate cumulative incidence curves illustrating transition probabilities according to the identified proteins. State occupation probabilities were estimated to quantify the probability of occupying each disease state at specific time points.

#### Single-Cell Analysis:

To exploratorily investigate the intestinal cellular expression profiles of the identified candidate genes, single-cell expression analysis was performed to evaluate the cell type–specific enrichment of the candidate protein-coding genes. A previously published human intestinal single-cell RNA-seq dataset (GSE134809) comprising inflamed and uninflamed ileal samples from 11 CD patients was analyzed.^21^ Data preprocessing and transformation were first performed using the “Seurat” package.^22^ The NormalizeData and ScaleData functions were then used to normalize and scale the RNA transcripts per million. Cell types were annotated using the “SingleR” package.^23^ A total of 33 694 genes across 103 856 cells were included in the analyses. To determine whether the candidate protein-coding genes were highly expressed in specific cell types, differential expression analysis was performed using the Wilcoxon rank-sum test. The expression levels of the candidate protein-coding genes in specific cell types were also compared between inflamed and uninflamed tissues using differential expression analysis. Differentially expressed genes were identified based on an average absolute |log2FC| greater than 0.25 and a false discovery rate–adjusted *P* < .05.

## Results

### Baseline Analysis on the Prevalence of Low Bone Density Among Patients with Inflammatory Bowel Disease

First, a baseline comparison of BMD and T scores was performed between patients with IBD—including UC, CD, and undetermined colitis—and healthy controls. A total of 445 064 participants were included at baseline, comprising 429 056 controls and 16 008 patients with IBD (Supplementary Figure 1). The median age was 56.6 ± 8.1 years; 54.5% of participants were female, and 94.1% were of White ethnicity (Supplementary Table 2). BMD and T-score assessments consistently showed lower values in patients with IBD than in healthy controls. Specifically, the mean T scores were −0.63 ± 1.16 for CD, −0.51 ± 1.17 for UC, −0.53 ± 1.22 for undetermined colitis, and −0.39 ± 1.22 for healthy controls. Similarly, the mean BMD values were 0.51 ± 0.13, 0.52 ± 0.13, 0.52 ± 0.14, and 0.54 ± 0.14, respectively (Supplementary Table 2 and Supplementary Figure 1). This pattern remained consistent in sex-specific subgroup analyses (Supplementary Figure 1).

### Population Characteristics of the Cohort Study

To conduct this cohort study, 52 953 participants with available Olink proteomics data were analyzed (Supplementary Figure 2). The cohort had a median age of 56.8 ± 8.2 years; 53.9% of participants were female, and 93.2% were White (Supplementary Table 3). Over a median follow-up of 13.6 years (IQR: 12.8-14.3), 155 cases of CD, 260 cases of UC, 1917 cases of undetermined colitis, and 2088 cases of osteoporosis were identified. Detailed characteristics of the participants are summarized in Supplementary Table 3.

### Identifying Proteins Associated with Inflammatory Bowel Disease and Osteoporosis

After adjustment for age, sex, ethnicity, BMI, TDI, education level, smoking status, alcohol consumption, physical activity, hypertension, lipidemias, and diabetes, Cox regression analysis was performed to identify plasma proteins associated with IBD or osteoporosis. Among these proteins, matrix metallopeptidase 12 (MMP12), hepatocyte growth factor (HGF), prostaglandin D2 synthase (PTGDS), ectonucleotide pyrophosphatase/phosphodiesterase family member 5 (ENPP5), regenerating family member 3 alpha (REG3A), transforming growth factor beta receptor 2 (TGFBR2), TNF receptor superfamily member 1A (TNFRSF1A), epidermal growth factor receptor (EGFR), growth differentiation factor 15 (GDF15), and uromodulin (UMOD) showed the strongest associations with IBD (including UC, CD, and undetermined colitis) (Figure 1). Among these proteins, ENPP5, EGFR, and UMOD were associated with a lower risk of IBD, whereas MMP12, HGF, PTGDS, REG3A, TGFBR2, TNFRSF1A, and GDF15 were associated with an increased risk. Meanwhile, adiponectin (ADIPOQ), insulin-like growth factor–binding protein 2 (IGFBP2), GDF15, sclerostin (SOST), EGFR, and matrix extracellular phosphoglycoprotein (MEPE) were significantly associated with osteoporosis. SOST, EGFR, and MEPE were associated with a lower risk of osteoporosis, whereas ADIPOQ, IGFBP2, and GDF15 were associated with an increased risk (Figure 1). Overall, 339 proteins remained significantly associated with IBD and 183 with osteoporosis, of which, 104 overlapped between the 2 conditions. The shared and condition-specific proteins are shown in Figure 1.

### Biological Function of Overlapped Proteins

Next, to investigate potential interactions among proteins associated with both IBD and osteoporosis, pathway enrichment analysis was performed on the 104 overlapped proteins. KEGG analysis revealed significant enrichment in pathways including “Intestinal immune network for IgA production,” “cytokine-cytokine receptor interaction,” “osteoclast differentiation,” and “interleukin-17 (IL-17) signaling pathway” (Figure 2). GO analysis was performed across BP, CC, and MF categories, highlighting terms such as “positive regulation of cell activation,” “positive regulation of leukocyte activation,” “cytokine activity,” and “cytokine receptor binding.” Transcription factor enrichment analysis using the transcription regulatory relationships unraveled sentence-based text mining database was performed to identify potential transcription factors targeting the 104 overlapped proteins. Among the candidates, BTB domain and CNC homolog 1 (BACH1), forkhead box M1 (FOXM1), tumor protein p63 (TP63), RELA proto-oncogene NF-κB subunit (RELA), and SRY-box transcription factor 2 (SOX2) showed the highest predictive relevance (Figure 2). Finally, protein–protein interaction (PPI) analysis revealed a network of interactions among the identified proteins, with EGFR, TNF, and IL-6 exhibiting the highest degree of connectivity.

### Machine Learning Identifies Proteins with High Predictive Importance

The predictive value of the 104 overlapped proteins for the subsequent co-development of IBD and osteoporosis in a healthy population was first evaluated. The proteins were ranked according to their association with both conditions, and the top 10 were selected to construct predictive models. The resulting modelsfor predicting IBD–osteoporosis, UC–osteoporosis, CD–osteoporosis, and undetermined colitis–osteoporosis achieved area under the curve (AUC) values greater than 0.70, indicating good predictive performance (Figure 3). SHAP (SHapley Additive exPlanations) analysis further illustrated the contributions of the top 10 proteins to each of the 4 models. A subset of proteins, including C-type lectin domain containing 5A (CLEC5A), IL-15, and IGFBP2, consistently demonstrated high predictive importance across multiple models.

The ability of these 104 proteins to predict osteoporosis risk specifically among patients with IBD was assessed. Models developed to predict osteoporosis in IBD, UC, CD, and undetermined colitis cohorts all demonstrated good predictive performance, as reflected by their AUC values. SHAP analysis again highlighted the strong predictive importance of the top 10 proteins within each patient subgroup (Figure 4). Furthermore, an overlapping set of high-performing proteins was identified across several models, underscoring their potential broader relevance to osteoporosis pathogenesis among patients with IBD.

### Multistate Modeling Indicates the Association Between Candidate Predictive Proteins and Inflammatory Bowel Disease–Osteoporosis Multimorbidity

To elucidate the stepwise involvement of highly predictive proteins in the transition from health to IBD and subsequently to IBD–osteoporosis multimorbidity, a multistate model analysis was performed. All top predictive proteins identified in the previous models were significantly associated with the development of IBD among initially healthy participants. However, only 8 proteins—plasminogen activator, urokinase receptor (PLAUR), TNF superfamily member 10a (TNFSF10A), serine peptidase inhibitor Kazal type 1 (SPINK1), major histocompatibility complex, class I, E (HLA_E), chromogranin A (CHGA), secreted phosphoprotein 1 (SPP1), TNF superfamily member 10b (TNFSF10B), and IGFBP2—were significantly associated with the progression from the transition from IBD to IBD–osteoporosis multimorbidity (Figure 4). These findings suggest that these 8 proteins may play key roles in the development of IBD-related osteoporosis.

### Single-Cell–Type Expression Analysis of Candidate Protein-Coding Genes

To explore the intestinal cellular expression profiles of these candidate protein-coding genes, single-cell**–**type expression analysis was performed to assess their cell type–specific enrichment of candidate protein-coding genes in intestinal tissue and their differential expression between inflamed and uninflamed regions. Twelve main cell types were identified through unsupervised clustering and annotation using classical marker gene, including enterocytes, epithelial cells, endothelial cells, fibroblasts, basal cells, goblet cells, glial cells, T cells, innate lymphoid cells, natural killer cells (NK cells), B cells, plasma cells, myeloid cells, and mast cells (Figure 5). Among the candidate genes, intestinal expression data were available for 5 genes: *TNFSF10A*, *SPINK1*, *CHGA*, *TNFSF10B*, and *IGFBP2*. Except for *CHGA* and *TNFSF10A*, the remaining genes showed significant cell type–specific enrichment in enterocytes (*SPINK1*), epithelial cells (*SPINK1,* *TNFSF10B*, and *IGFBP2*), endothelial cells (*IGFBP2*), and goblet cells (*SPINK1* and *IGFBP2*). Furthermore, most of these genes were significantly upregulated in their corresponding enriched cell types within inflamed tissues, consistent with the previous analytical findings (Supplementary Figure 3).

## Discussion

In this study, IBD–osteoporosis multimorbidity was investigated using plasma proteomic profiling to identify potential disease markers and elucidate the underlying mechanisms. Baseline comparisons showed that individuals with IBD had statistically lower calcaneal BMD and T scores than healthy controls, although the absolute differences were small. Using plasma proteomic data, proteins associated with IBD or osteoporosis were identified by Cox regression analysis and 104 overlapping proteins associated with both conditions were further detected. Pathway enrichment and PPI analyses were performed to characterize the main function of the overlapping proteins. Through machine learning and multistate modeling, 8 proteins with high predictive value for IBD–osteoporosis multimorbidity were identified and their intestinal cellular expression patterns were further characterized using single-cell expression analysis.

This targeted proteomic study, leveraging comprehensive plasma proteomic profiling in a large cohort, identified multiple proteins associated with IBD and osteoporosis. Cox regression analysis identified several proteins associated with the development of IBD, such as PTGDS, HGF, REG3A, MMP12, and TNFRSF1A. MMP12 has been reported to mediate basement membrane laminin degradation, macrophage transmigration, and disruption of intestinal tight-junction integrity—all of which are key processes in intestinal inflammation. These effects were attenuated in Mmp12-KO mice, which exhibited reduced macrophage infiltration, transmigration, and laminin degradation.^24^ Meanwhile, single-nucleotide polymorphisms in TNFRSF1A have been associated with CD, with the rs767455 GG genotype occurring more frequently among patients with CD.^25^ In another study, *HGF* was identified as a potential diagnostic biomarker for UC, with findings validated by ROC analysis, differential expression analysis, and a mouse model.^26^ Consistent with the present findings, previous bioinformatic and machine learning studies have also identified REG3A and PTGDS as candidate risk factors for UC progression.^27^^,^^28^ SOST, an inhibitor of the canonical Wnt-signaling pathway, is a well-established regulator of bone homeostasis^29^ and has been implicated in various skeletal disorders.^30^^-^^32^ Several preclinical and clinical studies have evaluated anti-SOST antibodies for the treatment of skeletal diseases and cancer.^33^ Additionally, IGFBP2 and ADIPOQ have been reported to influence osteogenic differentiation in bone marrow mesenchymal stem cells.^34^^,^^35^

Based on the proteins associated with IBD or osteoporosis, 104 overlapping proteins associated with both conditions were identified, and functional enrichment analysis of these overlapping proteins was subsequently performed. KEGG analysis revealed significant enrichment of cytokine–cytokine receptor interaction pathways, whereas GO analysis highlighted BPs related to leukocyte activation and proliferation. Consistent with the present findings, chronic inflammation—characterized by elevated levels of cytokines such as IL-1, IL-6, and TNF—can upregulate RANKL (ligand of receptor activator of the nuclear factor κB) and impair calcium and vitamin D absorption, thereby contributing to bone loss.^36^ However, true single-cell types involved in IBD–osteoporosis multimorbidity have not been well characterized. GO analysis suggested a potential role for leukocytes in this process. In a mouse model of hyperparathyroidism, intestinal TNF-positive T cells and Th17 cells were shown to egress via S1P-receptor-1 and migrate to the bone marrow, leading to bone loss. Similarly, in experimental colitis, leukocytes may migrate from the intestine to other organs, thereby contributing to extraintestinal manifestations.^37^ Notably, these overlapping proteins and classical pathways are closely associated with the pathogenesis of both conditions, providing a biological basis for understanding how chronic mucosal inflammation may contribute to systemic skeletal complications.

Machine learning was initially used to identify 8 proteins with high predictive value of IBD–osteoporosis multimorbidity. Importantly, subsequent incorporation of these prioritized markers into a multistate framework enabled characterization of the sequential trajectory of disease progression. Notably, IGFBP2 emerged as the strongest predictor. Serum IGFBP2 levels have been reported to be elevated in patients with IBD and to correlate positively with erythrocyte sedimentation rate and IL-1β.^38^ Similarly, serum IGFBP2 levels have been reported to be 1.8-fold higher in patients with osteoporosis than in matched controls, particularly among those with fractures.^39^ Another study linked IGFBP2 to LXR/RXR activation and acute-phase response signaling, pathways associated with an increased risk of fracture.^40^ These findings are consistent with the present findings, supporting a potential role for IGFBP2 in IBD–osteoporosis comorbidity and its value as a predictive biomarker. Single-cell expression analysis further showed that *IGFBP2* was enriched in colonic epithelial and goblet cells and was expressed at higher levels in inflamed than in noninflamed tissues, suggesting that these cell types may contribute to the development of osteoporosis in patients with IBD. Upon further clinical validation, this proteomic score may enable early risk stratification at the time of IBD diagnosis. This model may help identify patients at high risk of osteoporosis, thereby facilitating timely interventions to delay or prevent disease development.

This study identified candidate predictive proteins involved in the pathogenesis of IBD–osteoporosis multimorbidity through machine learning and multistate modeling. A key strength of the present study is the integration of high-throughput proteomic data with multimorbidity assessments in a large, community-based cohort with long-term follow-up. However, several limitations should be acknowledged. First, the use of broad ICD-10 diagnostic codes may have introduced misclassification of IBD and osteoporosis outcomes. Second, owing to data limitations, detailed clinical variables such as cumulative glucocorticoid exposure and localized disease activity were unavailable, leaving the possibility of residual confounding. Third, the generalizability of the findings to other populations may be limited because the cohort was derived from the UK Biobank and consisted predominantly of middle-aged and older individuals of European ancestry. Fourth, although the Olink platform provides broad proteomic coverage, it does not measure all circulating proteins, and protein expression in other relevant tissues could not be assessed. Finally, the candidate proteins and predictive models require further validation in external populations and experimental animal models.

## Conclusion

Using data from a population-based cohort, the proteomic landscape of IBD–osteoporosis multimorbidity was characterized, prospective associations of implicated proteins were identified, key proteins and biological pathways linking the 2 conditions were identified, and candidate biomarkers and hypothesis-generating mechanistic signals were highlighted.

## Supplementary Materials

Supplementary Material

## Figures and Tables

**Figure 1.**
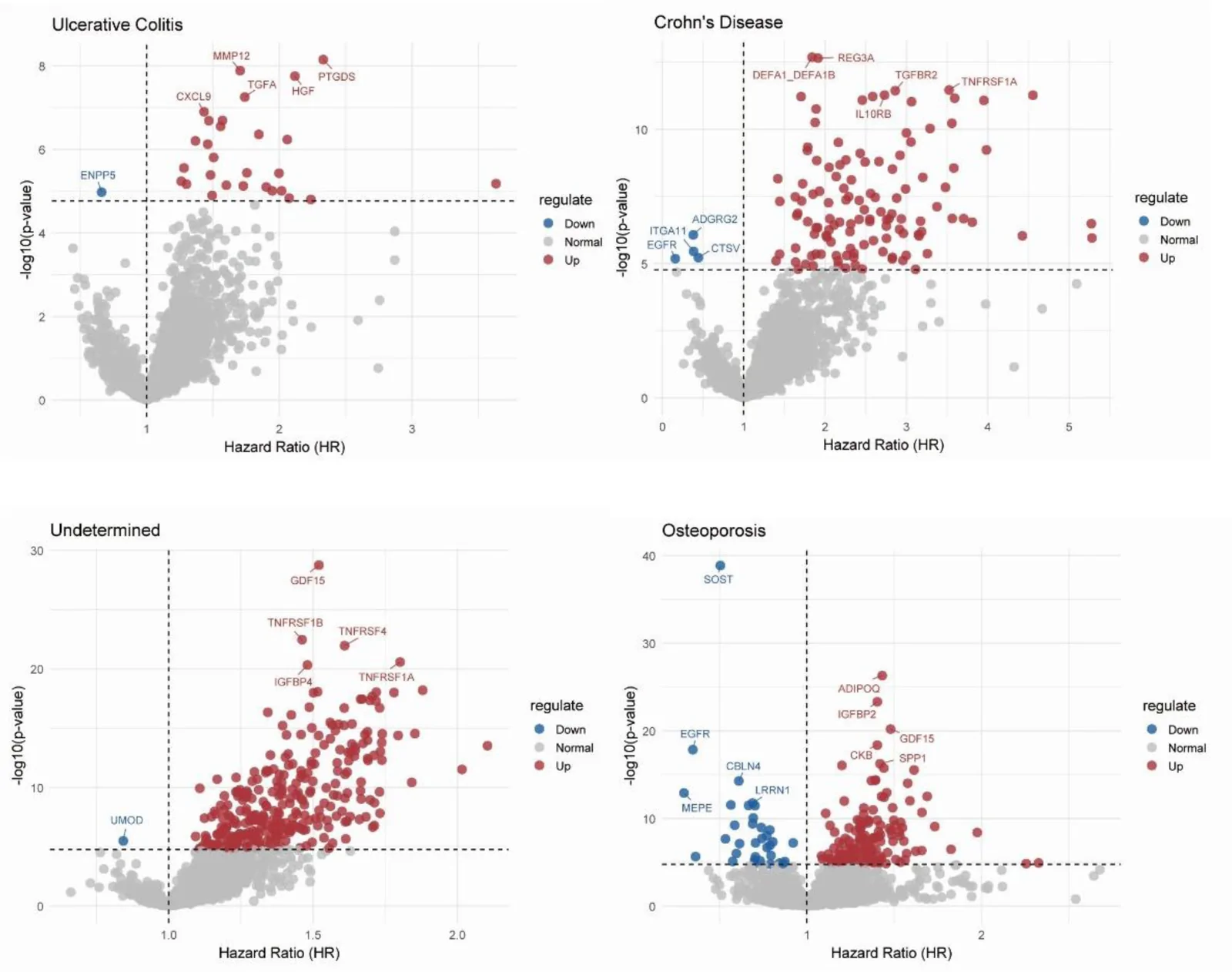
Proteins associated with incident inflammatory bowel disease and osteoporosis. Volcano plots displaying the HRs (*x-*axis) and statistical significance (−log10 of 2-sided *P-*values, *y-*axis) for the associations between standardized protein abundance with incident UC, CD, undetermined colitis, and osteoporosis. CD, Crohn’s disease; IBD, inflammatory bowel disease; HR, hazard ratio; UC, ulcerative colitis.

**Figure 2:**
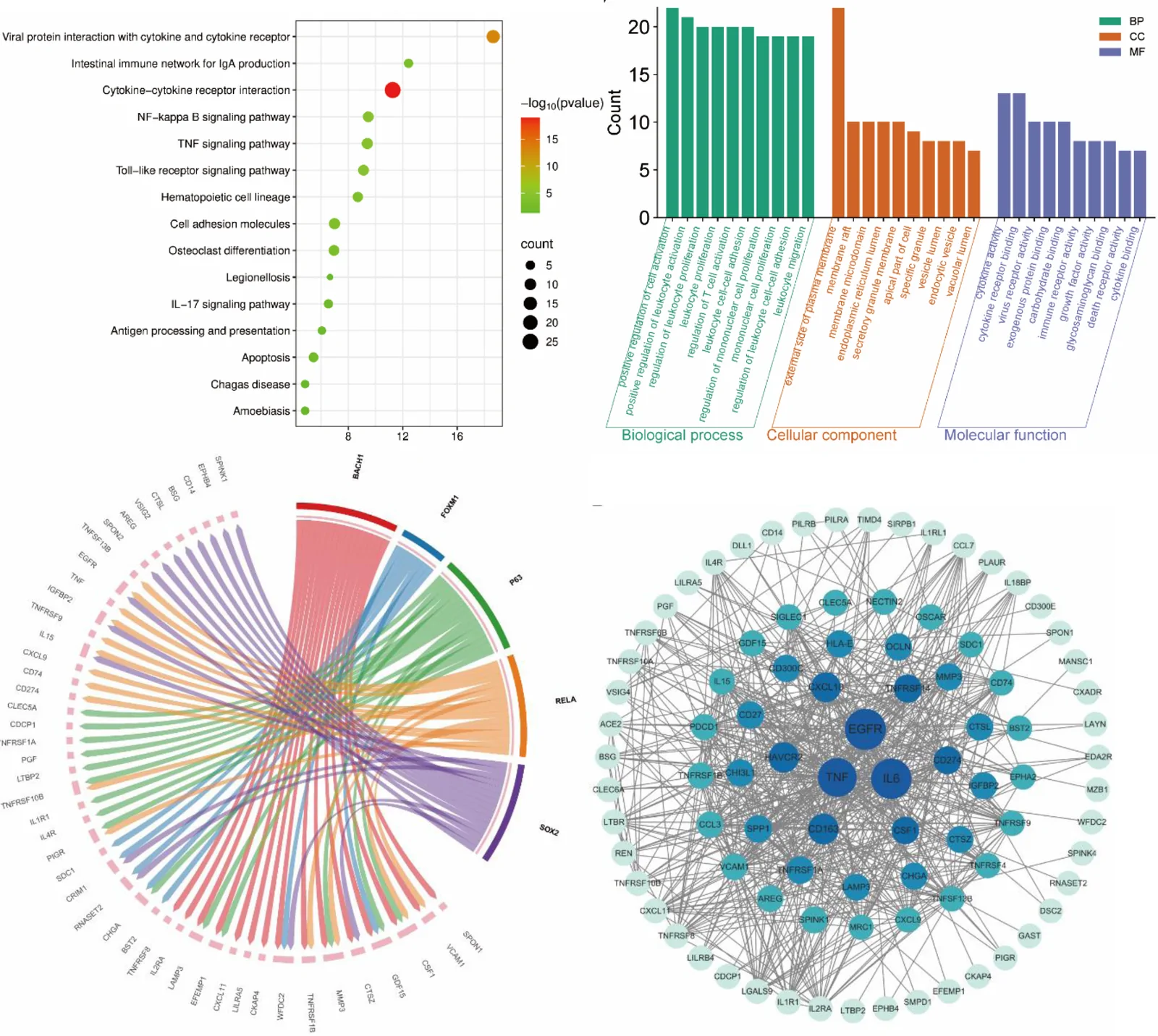
Functional characterization of comorbidity-associated proteins. The enrichment of overlapped proteins for IBD–osteoporosis comorbidity across KEGG biological processes. The enriched items for BP, CC, and MF, respectively, in the GO analysis. Results of the transcription factors enrichment analysis. The top 5 transcription factors that were significantly enriched are shown. Each enriched transcription factor target is connected to its corresponding target genes. A protein–protein interaction network revealed interactions between the identified proteins. BP, biological process; CC, cellular component; GO, gene ontology; IBD, inflammatory bowel disease; KEGG, Kyoto Encyclopedia of Genes and Genomes; MF, molecular function; PPI, protein–protein interaction.

**Figure 3:**
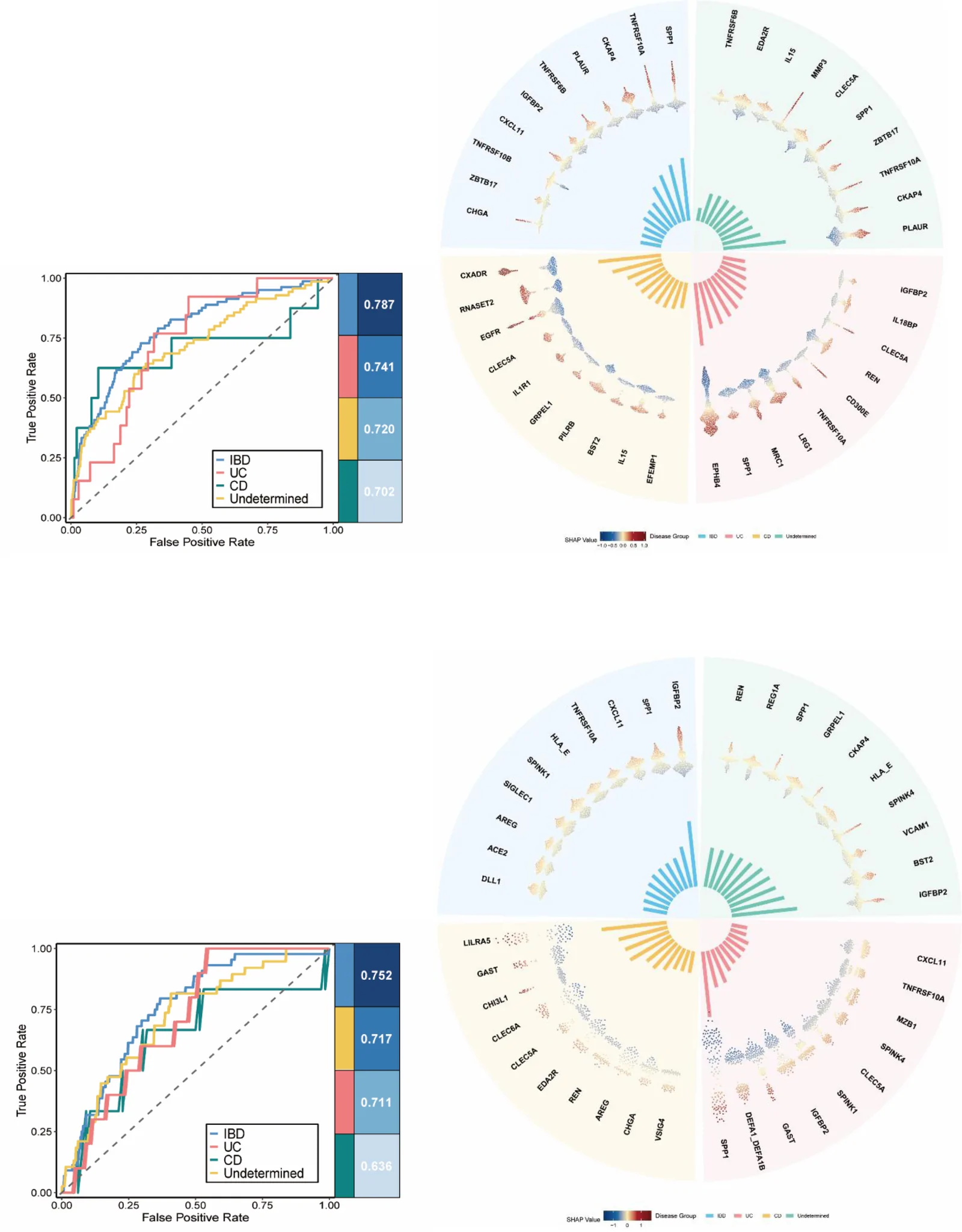
Identification of plasma proteomic features for IBD–osteoporosis multimorbidity. The significance of overlapped proteins in developing both IBD and osteoporosis among healthy and IBD patients was analyzed. Evaluation of 4 prediction models predicting IBD–osteoporosis, UC–osteoporosis, CD–osteoporosis, and undetermined colitis–osteoporosis comorbidity. Ranking of the importance of plasma protein features and SHAP visualization plot of selected proteins. CD, Crohn’s disease; IBD, inflammatory bowel disease; SHAP, SHapley Additive exPlanations; UC, ulcerative colitis.

**Figure 4:**
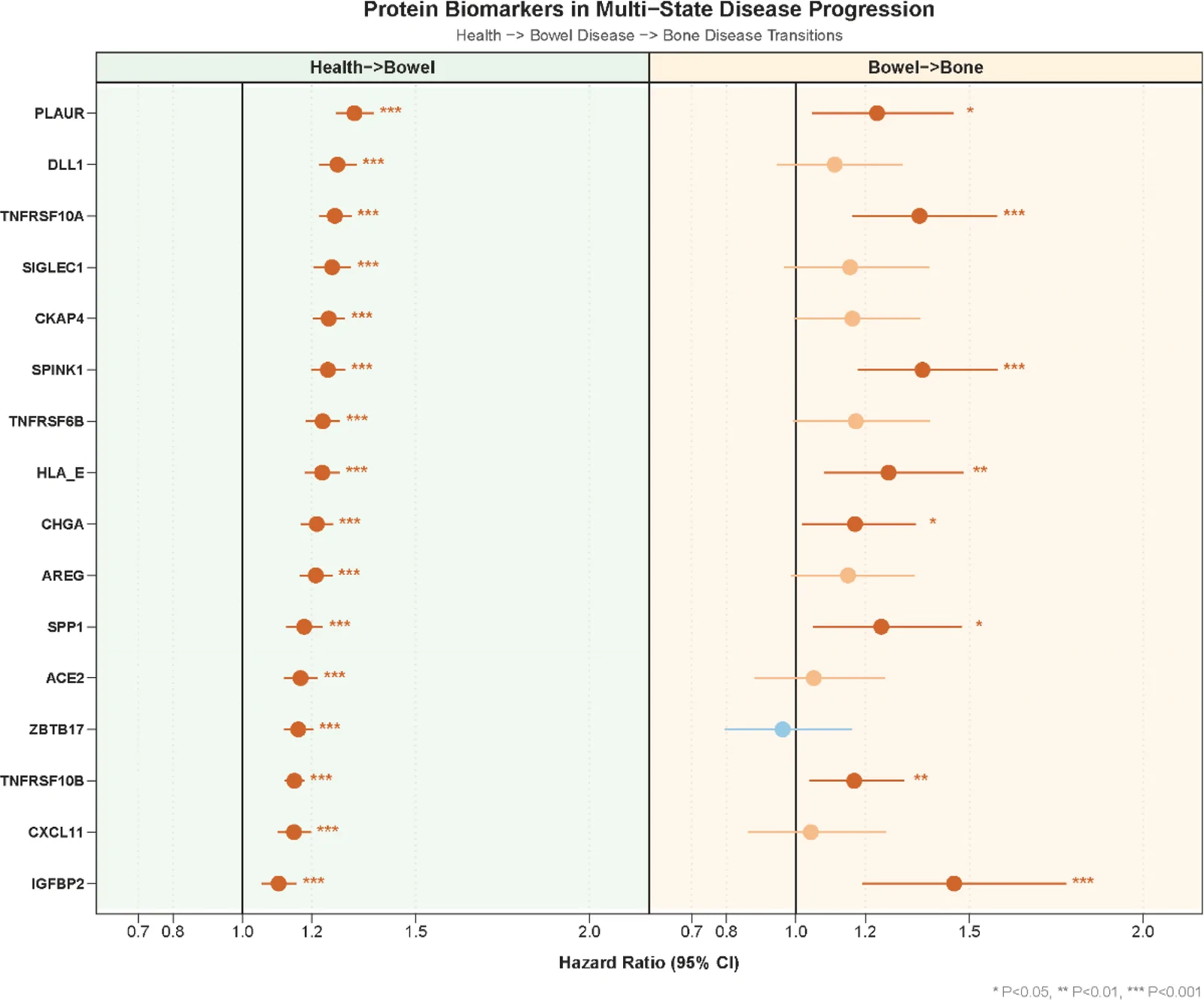
Multistate modeling indicates the association between high predictive power proteins and IBD–osteoporosis multimorbidity. The right panel represents the progression from the baseline “healthy” state to IBD, whereas the left panel show the transition from IBD to IBD–osteoporosis multimorbidity. ^*^P < .05, ^**^P < .01, and^ ***^P < .001. IBD, inflammatory bowel disease.

**Figure 5:**
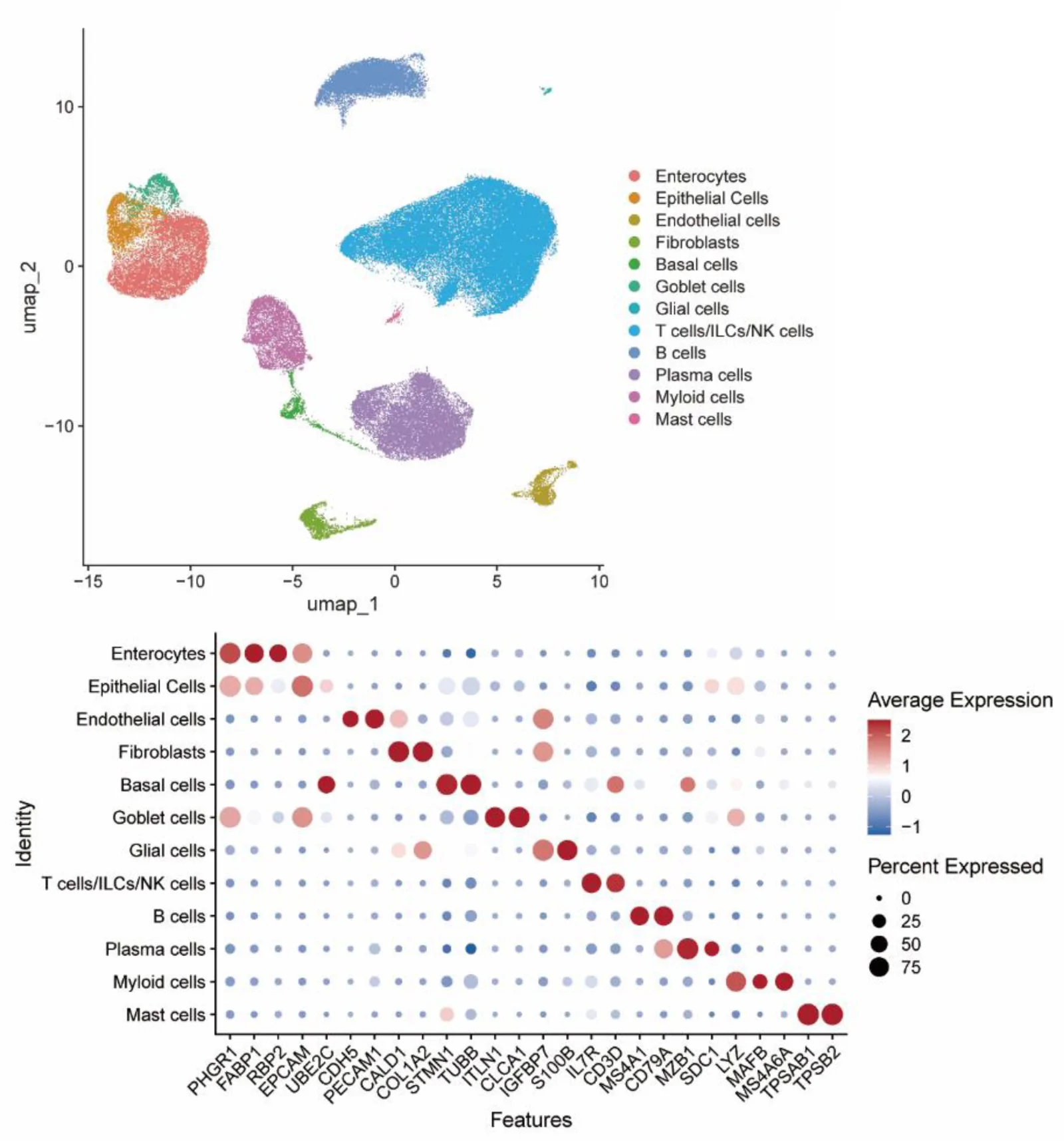
Single-cell**–**type expression in inflamed and uninflamed ileum. A total of 12 cell types were identified using graph-based clustering by the t-distributed stochastic neighbor embedding. Dot plots show the expression of representative marker genes in the indicated cell types. Dot color and size represent the average gene expression level and the percentage of expressing cells, respectively. Expression levels of high-performing protein-coding genes were compared between inflamed and uninflamed ileal tissues within the enriched cell types. The t-SNE plot shows the expression of high-performing protein-coding genes across different cell types in inflamed and uninflamed parts. The Wilcoxon rank-sum test was used for analysis. All tests were 2-sided and adjusted for multiple comparisons. Genes with an average log2 fold change > 0.25 and an FDR < 0.05 were considered significantly differentially expressed. FDR, false discovery rate; Log2FC, log2 fold change; t-SNE, t-distributed stochastic neighbor embedding.

## Data Availability

The data that support the findings of this study are available on request from the corresponding author.
