# Supplementary material for: Plasma Proteomic Profiling Identifies Biomarkers and Therapeutic Targets for Predicting Osteoporosis Risk in Patients with Inflammatory Bowel Disease
Source: Turk J Gastroenterol. 2026 Aug 4;37(10):1042–53. doi: 10.5152/tjg.2026.26320 (PMC13749452; doi:10.5152/tjg.2026.26320)
Supplement: Supplementary file 1 [file supplementary_material.pdf]

Supplementary Table 1. ICD-10 Codes for IBD and osteoporosis diagnosis.

Category	Disease Name	ICD-10 Code(s)
IBD	Crohn's diseases	K50
	Ulcerative colitis	K51
	Undetermined	K52
Osteoporosis	Osteoporosis	M80, M81

IBD, Inflammatory Bowel Disease; ICD-10, International Classification of Diseases-10.

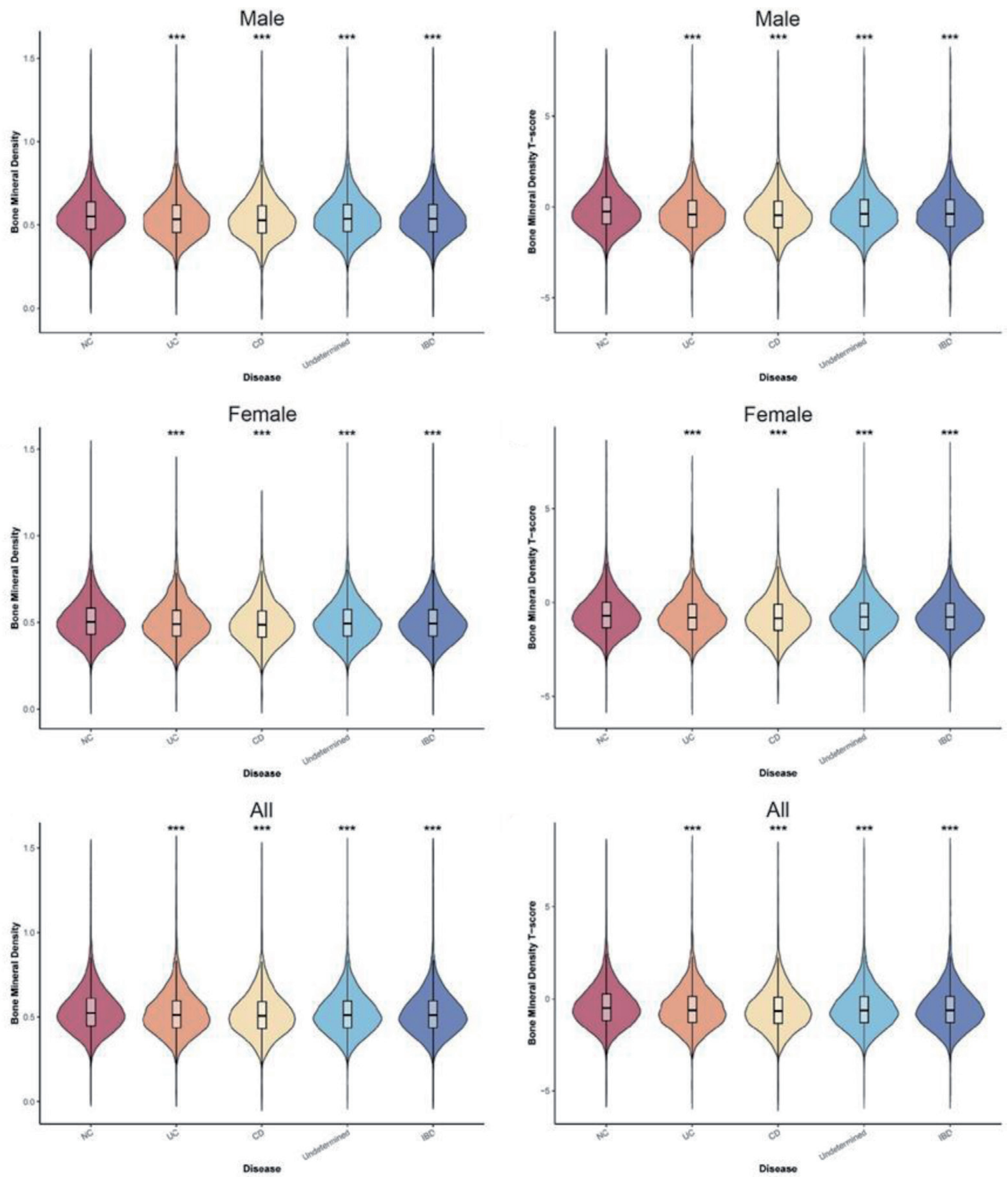

Supplementary Figure 1. Bone mineral density evaluation among IBD patients and controls. The left panels and right panels show the evaluation of bone mineral density and T-scores, respectively, among controls and patients with UC, CD, undetermined colitis, and overall IBD. Analyses were performed across different sex subgroups as well as among all participants combined. ***P < 0.001. IBD, inflammatory bowel disease; UC, ulcerative colitis; CD, crohn's disease.

Supplementary Table 2. Characteristics of the baseline analysis.

	CD (N=1,503)	UC (N=2,968)	Undetermined (N=11,537)	NC (N=429,056)	Overall (N=445,064)
Sex					
Female	840 (55.9%)	1,524 (51.3%)	7,077 (61.3%)	232,902 (54.3%)	242,343 (54.5%)
Male	663 (44.1%)	1,444 (48.7%)	4,460 (38.7%)	196,154 (45.7%)	202,721 (45.5%)
Age					
Mean (SD), years	56.5 (8.06)	57.7 (7.75)	57.6 (7.95)	56.5 (8.08)	56.6 (8.08)
Ethnic					
Other	47 (3.1%)	135 (4.5%)	615 (5.3%)	25,422 (5.9%)	26,219 (5.9%)
White	1,456 (96.9%)	2,833 (95.5%)	10,922 (94.7%)	403,634 (94.1%)	418,845 (94.1%)
TDI					
Mean (SD)	-1.22 (3.08)	-1.43 (2.95)	-0.873 (3.24)	-1.37 (3.05)	-1.36 (3.05)
Education level					
College and above	397 (26.4%)	839 (28.3%)	3,063 (26.5%)	138,846 (32.4%)	143,145 (32.2%)
High school and below	1,106 (73.6%)	2,129 (71.7%)	8,474 (73.5%)	290,210 (67.6%)	301,919 (67.8%)
Smoking					
Current	225 (15.0%)	207 (7.0%)	1,438 (12.5%)	44,073 (10.3%)	45,943 (10.3%)
Never	678 (45.1%)	1,445 (48.7%)	5,894 (51.1%)	237,249 (55.3%)	245,266 (55.1%)
Previous	600 (39.9%)	1,316 (44.3%)	4,205 (36.4%)	147,734 (34.4%)	153,855 (34.6%)
Alcohol drinking					
Current	1,343 (89.4%)	2,686 (90.5%)	10,078 (87.4%)	395,359 (92.1%)	409,466 (92.0%)
Never	76 (5.1%)	147 (5.0%)	733 (6.4%)	18,777 (4.4%)	19,733 (4.4%)
Previous	84 (5.6%)	135 (4.5%)	726 (6.3%)	14,920 (3.5%)	15,865 (3.6%)
BMI					
Mean (SD)	26.8 (4.61)	27.3 (4.58)	28.0 (5.25)	27.4 (4.72)	27.4 (4.74)
Physical activity					
High	795 (52.9%)	1,598 (53.8%)	6,273 (54.4%)	232,950 (54.3%)	241,616 (54.3%)
Moderate	441 (29.3%)	883 (29.8%)	3,407 (29.5%)	135,337 (31.5%)	140,068 (31.5%)
Low	267 (17.8%)	487 (16.4%)	1,857 (16.1%)	60,769 (14.2%)	63,380 (14.2%)
Hypertension, n (%)	420 (27.9%)	861 (29.0%)	3,938 (34.1%)	113,689 (26.5%)	118,908 (26.7%)
Lipidaemias, n (%)	314 (20.9%)	801 (27.0%)	3,838 (33.3%)	109,018 (25.4%)	113,971 (25.6%)
Diabetes, n (%)	95 (6.3%)	218 (7.3%)	1,040 (9.0%)	21,690 (5.1%)	23,043 (5.2%)
BMD T-score					
Mean (SD)	-0.632 (1.16)	-0.505 (1.17)	-0.530 (1.22)	-0.391 (1.22)	-0.396 (1.22)
BMD					
Mean (SD)	0.510 (0.129)	0.524 (0.131)	0.521 (0.136)	0.537 (0.136)	0.537 (0.136)

CD, Crohn's disease; UC, ulcerative colitis; NC, normal control; TDI, townsend deprivation index; BMI, body mass index; BMD bone mineral density; SD, standard deviation.

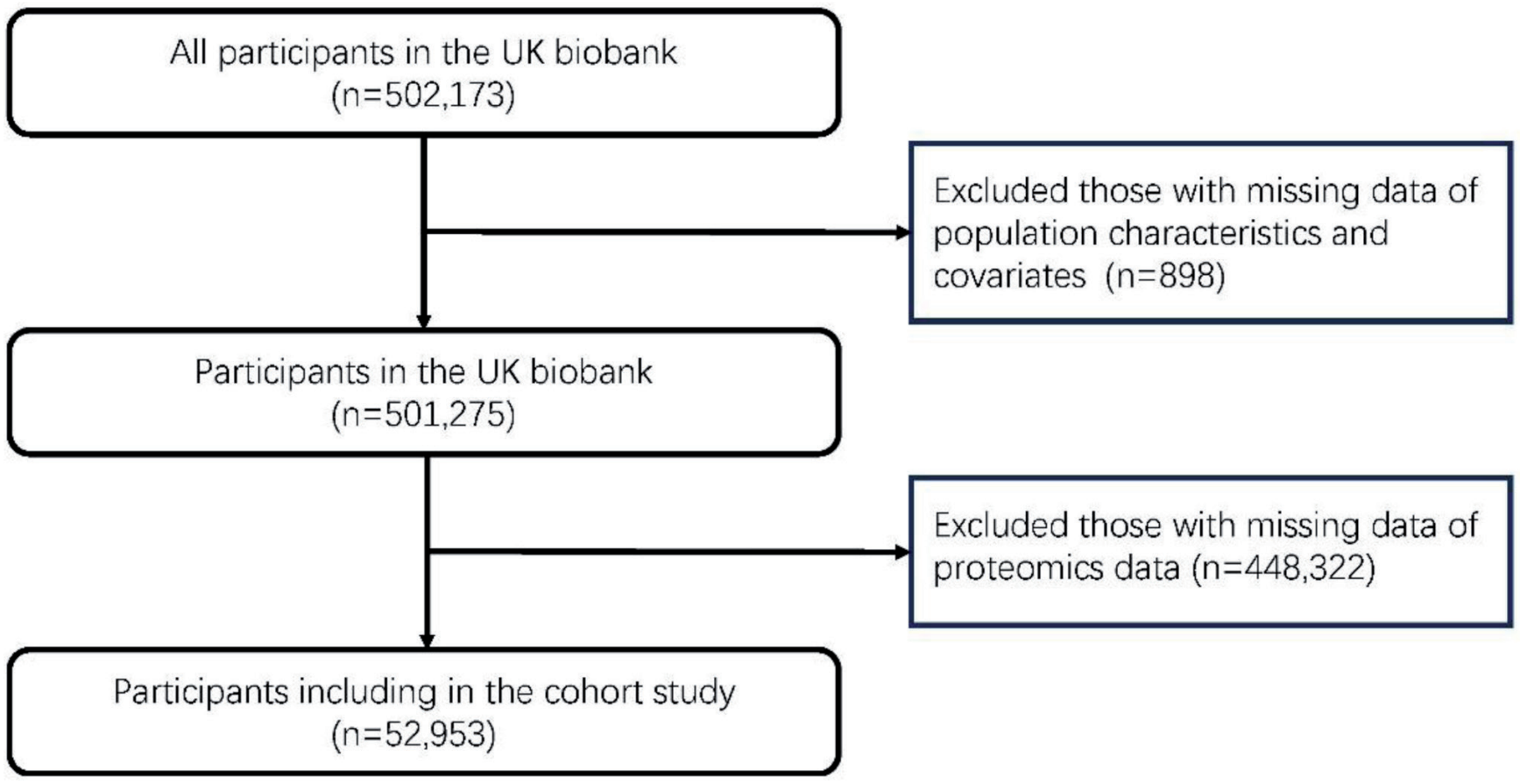

Supplementary Figure 2. Flow chart of the cohort study.

Supplementary Table 3. Baseline characteristics of the cohort study.

	Female (N=28,546)	Male (N=24,407)	Overall (N=52,953)
Age			
Mean (SD), years	56.6 (8.10)	57.0 (8.33)	56.8 (8.21)
Ethnic			
Other	1891 (6.6%)	1694 (6.9%)	3585 (6.8%)
White	26655 (93.4%)	22713 (93.1%)	49368 (93.2%)
TDI			
Mean (SD)	-1.23 (3.12)	-1.13 (3.25)	-1.18 (3.18)
Alcohol drinking			
Current	25682 (89.9%)	22704 (93.1%)	48386 (91.4%)
Never	1751 (6.1%)	755 (3.1%)	2506 (4.7%)
Previous	1113 (3.9%)	948 (3.9%)	2061 (3.9%)
Smoking			
Current	2544 (8.9%)	3056 (12.5%)	5600 (10.6%)
Never	17018 (59.6%)	11846 (48.5%)	28864 (54.5%)
Previous	8984 (31.5%)	9505 (38.9%)	18489 (34.9%)
Education level			
College and above	8750 (30.7%)	8173 (33.5%)	16923 (32.0%)
High school and below	19796 (69.3%)	16234 (66.5%)	36030 (68.0%)
Physical activity			
High	15800 (55.3%)	13028 (53.4%)	28828 (54.4%)
Moderate	8827 (30.9%)	7516 (30.8%)	16343 (30.9%)
Low	3919 (13.7%)	3863 (15.8%)	7782 (14.7%)
BMI			
Mean (SD)	27.1 (5.15)	27.8 (4.17)	27.4 (4.74)
Hypertension, n (%)	6,876 (24.1%)	8,048 (33.0%)	14,924 (28.2%)
Hyperlipidemia, n (%)	6,197 (21.7%)	7,986 (32.7%)	14,183 (28.2%)
Diabetes, n (%)	1,124 (3.9%)	1,893 (7.8%)	3,017 (5.7%)

TDI, townsend deprivation index; BMI, body mass index; SD, standard deviation.

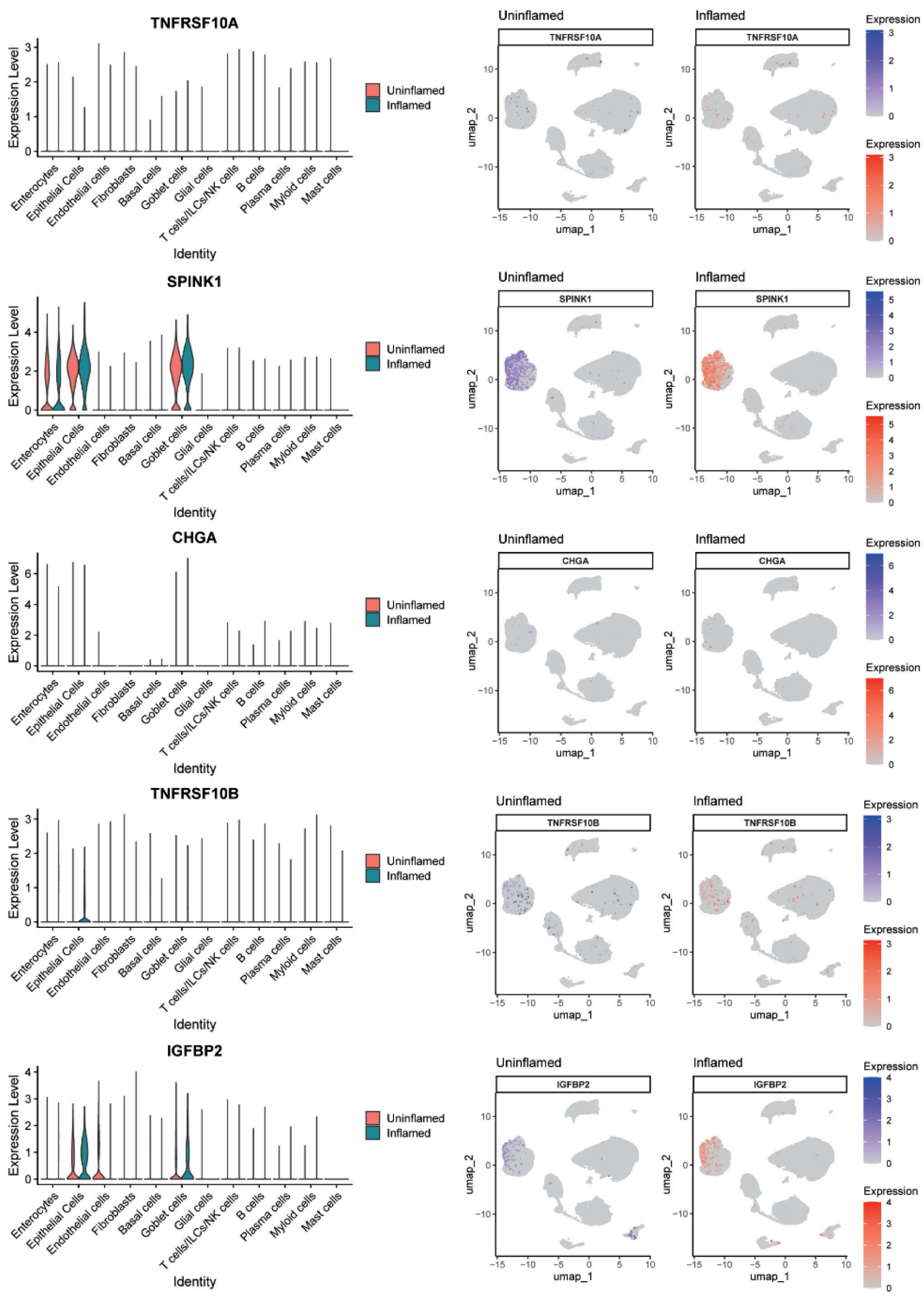

Supplementary Figure 3. Single-cell type expression in inflamed and uninflamed ileum. The average gene expression and percentage of cells expressed are shown by dot color and size, respectively. Comparison of high predictive power protein-coding gene expression levels between inflamed and uninflamed parts in enriched cell types. The t-SNE plot shows the expression of high predictive power protein-coding genes across different cell types in inflamed and uninflamed parts. The Wilcoxon rank-sum test was used for analysis. All tests were two sided and adjusted for multiple comparisons. Log2FC > 0.25 and an FDR < 0.05 was considered as significant. t-SNE, t-distributed stochastic neighbor embedding; Log2FC, log2 fold change; FDR, false discovery rate.
